# Intensive weight gain therapy in patients with anorexia nervosa results in improved serum tartrate-resistant acid phosphatase (TRAP) 5a and 5b isoform protein levels

**DOI:** 10.1007/s40519-019-00776-8

**Published:** 2019-09-17

**Authors:** Christina Patlaka, Bojan Tubic, Pernilla Lång, Staffan Paulie, Diana Swolin-Eide, Per Magnusson, Göran Andersson

**Affiliations:** 1 Division of Pathology, Department of Laboratory Medicine, Karolinska Institutet, Huddinge, Sweden; 2 Department of Orthopaedics, Institute of Clinical Sciences, Sahlgrenska University Hospital, Sahlgrenska Academy, University of Gothenburg, Göteborg, Sweden; 3 Mabtech AB, Nacka Strand, Sweden; 4 Department of Pediatrics, Institute of Clinical Sciences, Sahlgrenska Academy, University of Gothenburg, Gothenburg, Sweden; 5 The Queen Silvia Children’s Hospital, Sahlgrenska University Hospital, Gothenburg, Sweden; 6 Department of Clinical Chemistry, and Department of Clinical and Experimental Medicine, Linköping University, Linköping, Sweden

**Keywords:** Tartrate-resistant acid phosphatase, Acp5, Anorexia nervosa, Osteoclasts, Bone, Biomarker

## Abstract

**Aim:**

Tartrate-resistant acid phosphatase (TRAP) exists as isoforms 5a and 5b. TRAP 5a is a biomarker of chronic inflammation and influences adipose tissue and 5b associates with bone metabolism/pathologies. The aim was to investigate the association of serum TRAP 5a/5b isoforms with fat and bone markers and anthropometric parameters in patients with anorexia nervosa (AN) during weight gain therapy.

**Methods:**

Twenty-five Swedish female AN patients, age 16–24 years, were treated for 12 weeks with a high-energy diet with six meals daily. Serum TRAP 5a/5b, markers of fat/glucose metabolism, markers of bone resorption and formation were measured. Parameters of bone and body composition were assessed by dual-energy X-ray absorptiometry and peripheral quantitative computed tomography.

**Results:**

BMI increased from median 15.4 kg/m^2^ to 19.0 kg/m^2^, *p* < 0.0001. TRAP 5a and 5a/5b ratio increased but TRAP 5b decreased during the study. TRAP Δ5a and Δ5b correlated with Δinsulin and Δadiponectin, respectively. TRAP 5b correlated with trabecular density at start but not at week 12. At 12 weeks, TRAP 5b correlated with CTX, and Δ decrease in TRAP 5b correlated to Δ increase in bone-specific alkaline phosphatase.

**Conclusions:**

This clinical interventional study resulted in increased BMI in patients with AN. The decreased TRAP 5b protein levels confirm a role for TRAP 5b as a marker of bone resorption, whereas increased TRAP 5a seemed to derive from systemic changes in bone as well as metabolic changes. The combined detection of TRAP 5a and TRAP 5b in serum could be an indicator of improved bone metabolism.

**Level of evidence:**

Level III, prospective interventional cohort study.

**Electronic supplementary material:**

The online version of this article (10.1007/s40519-019-00776-8) contains supplementary material, which is available to authorized users.

## Introduction

Anorexia nervosa (AN) is a severe psychiatric disorder predominantly affecting female adolescents. In most cases, teenagers will gradually recover from the illness, although AN is known for its severe complications [1]. The prevalence of AN is approximately 1% among 17-year-old Swedish girls [2]. Many patients recover after treatment as outpatients, while some patients require hospitalization. A novel intensive nutrition therapy that initially starts with an extreme high caloric intake has recently and successfully been implemented for hospitalized AN patients resulting in significant increase in body mass index (BMI), weight and fat mass [3].

Peak bone mass is mostly acquired during early adulthood, which makes youth the best time to invest in bone health [4]. Reduced bone mineral density (BMD) is a common finding in patients with AN, which is a sign of impaired bone health [5]. AN treatments, such as intensive nutrition therapy, increase BMD, bone mineral content (BMC) and biochemical parameters for bone formation such as bone-specific alkaline phosphatase (BALP) and osteocalcin [6]. However, restoring bone health in AN patients is a slow process [7] resulting in long-term increased fracture risk [8, 9].

Tartrate-resistant acid phosphatase (TRAP; EC 3.1.3.2; Acp5; TRAcP) is a novel marker of adipose tissue regulation as well as a classical marker of osteoclasts and bone resorption. TRAP consists of two isoforms, TRAP 5a and TRAP 5b, which differ in structure, phosphatase activity [10], localization and most importantly biomarker potential [11]. TRAP 5a has been shown to be associated with inflammatory conditions such as obesity [12], overweight [13, 14], sarcoidosis [15] and rheumatoid arthritis [16, 17]. Conversely, TRAP 5b has been used as a marker of bone resorption [18, 19], chronic kidney disease [20], coronary atherosclerosis [21] and has been proposed as a marker of bone cancer metastasis [22]. With regard to AN patients, only TRAP 5b has been studied with mixed results. TRAP 5b was reportedly upregulated in one study [23] but unchanged in another investigation [24].

The TRAP isoforms 5a and 5b have previously been shown to be associated with mechanisms regulating both adipose tissue and bone remodeling. Since both systems are involved in the pathophysiology of AN, this study was designed to elucidate if the circulating levels of TRAP 5a and/or 5b could contribute with further information regarding the cellular response, in adipose tissue and/or bone remodeling, to weight gain therapy in young female patients with AN.

## Methods

### Study population

The present study took place between January 2012 and July 2014 and patients were adolescents with severe AN admitted to the Queen Silvia Children’s Hospital, Göteborg, Sweden. Patients fulfilling inclusion criteria were offered the 12 week treatment aiming to restore body weight and normalize eating behavior via a structured behavioral program [3]. Twenty-five female participants, age 16–24 years, fulfilled inclusion criteria being diagnosed with AN according to the Diagnostic and Statistical Manual of Mental Disorders IV [25]. Exclusion criteria included diabetes mellitus, inflammatory bowel disease, or life-threatening physical conditions demanding care at internal medicine department. The study was approved by the regional research ethics committee at the University of Gothenburg 720-11. Informed consent and assent were obtained from all study participants and in cases of minor subjects (< 18 years of age) also from their parents. Of the 25 patients, 2 discontinued the study because they did not want to participate in the 12-week program and one because diagnosis was reconsidered and, thus, the final number of subjects included in the current study was 22 (Supplementary Fig. 1).

### Study design

Study design was as described by Pettersson et al. [3]. Energy and nutrient intake was planned, served and supervised for all patients during 12 weeks and 24 h per day. Patients underwent nutritional rehabilitation for 12 weeks, with an extra high-energy diet, starting at median 75 kcal/kg/day and gradually declining to 48 kcal/kg/day during the study [3]. In addition, from day two, all patients were served 3 × 200 ml of high-energy liquid nutritional supplements (1.5 kcal/ml) together with snacks and night meal. As the patients gained weight, liquid supplements were removed from the menu. The mean nutrient intakes at week 1 and 12 were: protein 17 E% and 17 E%; fat 34 E% and 33 E%; and carbohydrate 48 E% and 48 E%, respectively. Subjects increased in median 9.9 kg (5.5–17.0 kg) during the study.

### Measurement of physiological and biochemical parameters

Measurement of physiological and biochemical parameters, baseline cohort characteristics and biochemical parameters at start and 12 weeks were performed as reported elsewhere [3, 6].

### Assessment of BMD and body composition

Fat mass, lean body mass as well as BMD and BMC were measured with dual-energy X-ray absorptiometry (DXA; Lunar Prodigy, GE Lunar Corp., Madison, WI) for total body (TB), lumbar spine (L1-L4), left arm and left leg. The peripheral quantitative computed tomography (pQCT) measurements were performed on the left tibia at 4% and 66% of the tibia length using an XCT 2000 (Stratec Medizintechnik GmbH, Pforzheim, Germany) with software version 6.00 as reported elsewhere [26]. The left calcaneal BMD and BMC were measured using DXA and laser (DXL) Calscan technique (Demetech AB, Solna, Sweden), where the measurement by DXA is combined with a laser measurement of the total calcaneal thickness [27]. All DXA, DXL and pQCT measurements were done by the same staff as previously reported [3].

### Serum pre-treatment for ELISA and standard curve dilution of recombinant TRAP

Samples were pre-treated in 1 volume 1 M glycine pH 2.3 at 37 °C for 1 h and then 1 volume of 1 M Tris–HCl, pH 8.3 was added. Samples were then diluted 1:1 in ELISA diluent buffer (Mabtech AB, Nacka, Sweden). Recombinant TRAP 5a standards were diluted in 1 M glycine pH 2.3, 1 M Tris–HCl, pH 8.3, and then in ELISA diluent buffer, ratio 1:1:4.

### Monoclonal antibody (mAb) and TRAP 5a separation from TRAP 5b

The mouse monoclonal antibody 46 (Mabtech) recognizes TRAP 5a [12] and, thus, plates coated with mAb 46 were used for quantifying and clearing of TRAP 5a in serum samples. MAb 25.44 (Mabtech) recognizes both TRAP 5a and TRAP 5b; thus, it was used after pre-incubation of samples with mAb 46 to capture and quantify the remaining fraction of TRAP that should only contain TRAP 5b.

### TRAP 5a and TRAP 5b sandwich ELISA

For capturing of TRAP 5a, samples and recombinant TRAP 5a standards were incubated at 4 °C overnight in 96-well half-volume ELISA plates (Costar, Chicago, IL) and coated with 5 μg/ml mAb 46 at 4 °C overnight. The samples, depleted of TRAP 5a, were thereafter transferred to 96-well half-volume ELISA plates (Costar) and coated with 5 μg/ml mAb 25.44 at 4 °C overnight, for capturing of TRAP 5b. Recombinant TRAP 5a standards (0–4 ng/ml) were additionally added to the mAb 25.44 plate. After incubation for 2 h at room temperature, plates were washed 3 times in TBST (25 mM Tris pH 7.6, 150 mM NaCl, 0.1% Tween 20) followed by incubation with 0.25 μg/ml biotinylated detection mAb 12.56 for 1 h at room temperature. After washing three times in TBST, plates were incubated with streptavidin–horseradish–peroxidase (Mabtech) 1:1000 for 30 min and washed 3 times in TBST. Finally, plates were developed using K-Blue Substrate (TMB; Neogen, Lansing, MI) for 20 min and stopped using 50 µl 1 M H_2_SO_4_ after which absorbance was measured at 450 nm in BioTek’s PowerWave HT microplate spectrophotometer (BioTek, Winooski, VT). Quantification of TRAP 5a was done using the TRAP 5a standard curve on the mAb 46-coated plate. Quantification of TRAP 5b was done using the newly added TRAP 5a standards and the samples depleted of TRAP 5a on the plate coated with mAb 25.44. The transferred standards of TRAP 5a from mAb 46 to mAb 25.44 plate were used to correct for any potential carryover of TRAP 5a from plate mAb 46 to plate mAb 25.44.

### Statistical analysis

For the analysis of TRAP 5a and TRAP 5b before and after 12 weeks, samples were compared using Wilcoxon signed-rank test. Correlation analysis of TRAP 5a and TRAP 5b to physiological and biochemical parameters was performed using Spearman’s rank correlation as not all parameters were normally distributed as evaluated with Shapiro–Wilk normality test. A *p* value of < 0.05 was considered statistically significant for all analyses. All statistical analysis was performed using the GraphPad Prism 6 software (GraphPad Software, Inc., La Jolla, CA).

## Results

### Serum TRAP 5a and TRAP 5b are changed in opposite directions after weight gain therapy

The initial weight of the subjects was in median (minimum–maximum) 44.3 kg (36.3–50.6 kg), which increased to 54.3 kg (44.0–61.4), *p* < 0.0001, at week 12. Subjects gained in median 9.9 kg (5.5–17.0 kg) during the study period (22% of initial weight). BMI increased from median 15.4 kg/m^2^ (13.4–17.3 kg/m^2^) to 19.0 kg/m^2^ (16.2–20.6 kg/m^2^), *p* < 0.0001. Fat mass percentage, measured by DXA, increased from median (minimum–maximum) 11.4% (4.4–24.8%) to 26.7% (16.9–39.8%), *p* < 0.0001 [28].

**Fig. 1:**
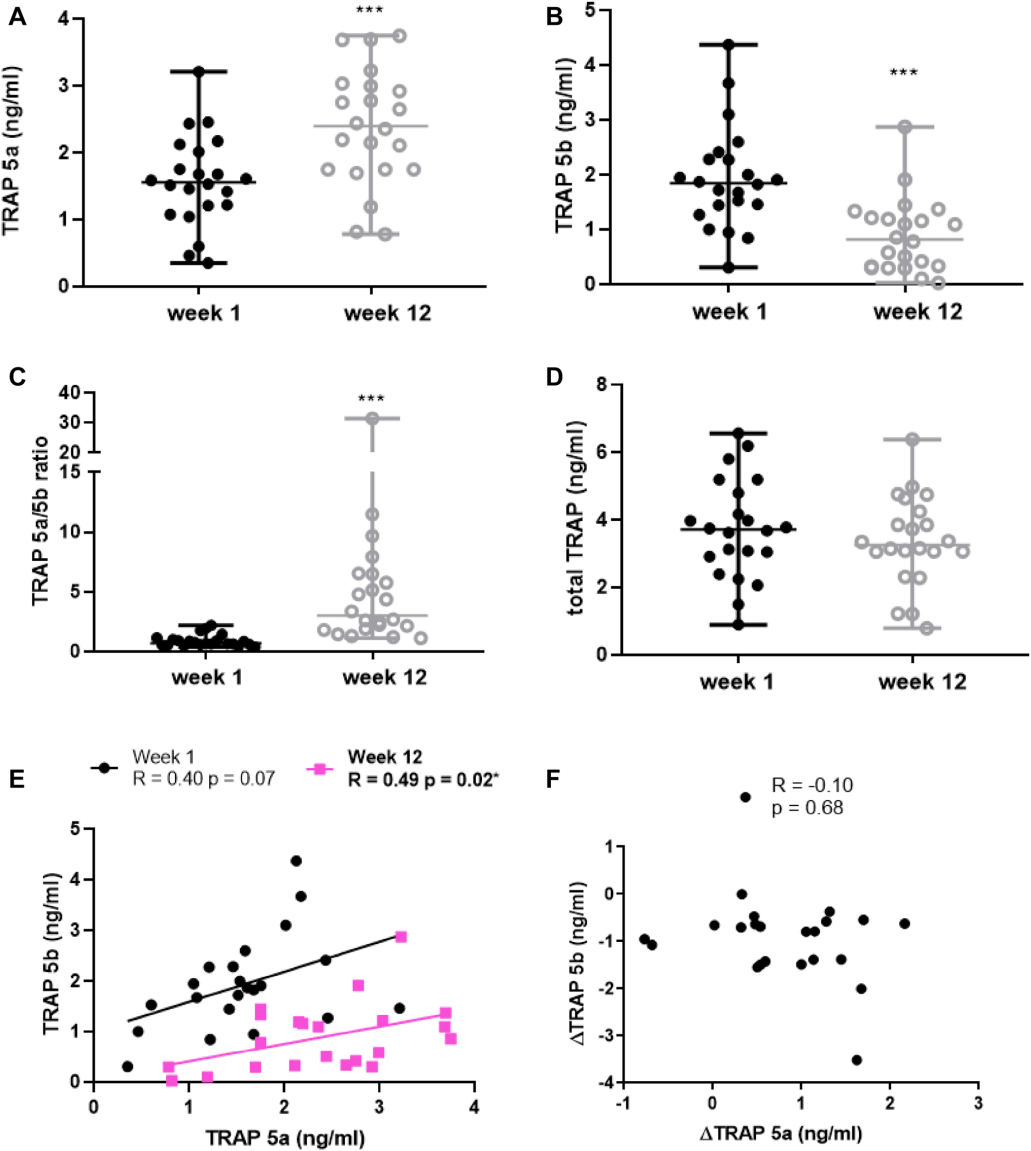
TRAP isoform profile in anorexia nervosa patients during intensive weight gain therapy. **a** Serum TRAP 5a protein. **b** Serum TRAP 5b protein. **c** Ratio of TRAP 5a/5b protein. **d** Serum total TRAP (TRAP 5a and 5b) protein. **e** Correlation of TRAP 5a and 5b protein weeks 1 and 12. **f** Correlation of ΔTRAP (weeks 12–week 1) 5a and Δ5b. *N* = 22 in all analysis. Values are shown as median ± range in all analysis. ***p < 0.001

At the study start, serum TRAP 5a levels were 1.6 (0.35–3.21) ng/ml [median (minimum–maximum)], while after 12 weeks of intensive weight gain therapy, TRAP 5a levels increased significantly to 2.4 (0.78–3.75) ng/ml, *p* = 0.0002 (Fig. 1a). On the contrary, serum TRAP 5b decreased from 1.85 (0.31–4.37) ng/ml at the study start to 0.82 (0.03–2.87) ng/ml, *p* < 0.0001, after 12 weeks (Fig. 1b). The TRAP 5a/TRAP 5b ratio increased significantly from 0.71 (0.39–2.20) at the study start to 3.02 (1.12–31.42) at week 12, *p* < 0.0001 (Fig. 1c). Total TRAP levels (TRAP 5a and 5b) did not change significantly over the study period, *p* = 0.08 (Fig. 1d); total TRAP levels at study start were 3.71 (0.90–6.56) ng/ml and 3.25 (0.79–6.38) ng/ml on week 12.

At the study start, correlation analysis demonstrated that serum TRAP 5a had a tendency for a positive correlation with serum TRAP 5b (*R* = 0.40, *p* = 0.07) and at week 12 there was a positive correlation between TRAP 5a and 5b (*R* = 0.49, *p* = 0.02) (Fig. 1e). No association was observed between the delta (Δ, change between weeks 1 and 12) values for TRAP 5a and 5b (Fig. 1f).

### TRAP 5a and TRAP 5b, as well as changes in their levels, do not correlate to weight anthropometric parameters [29]

**Fig. 2:**
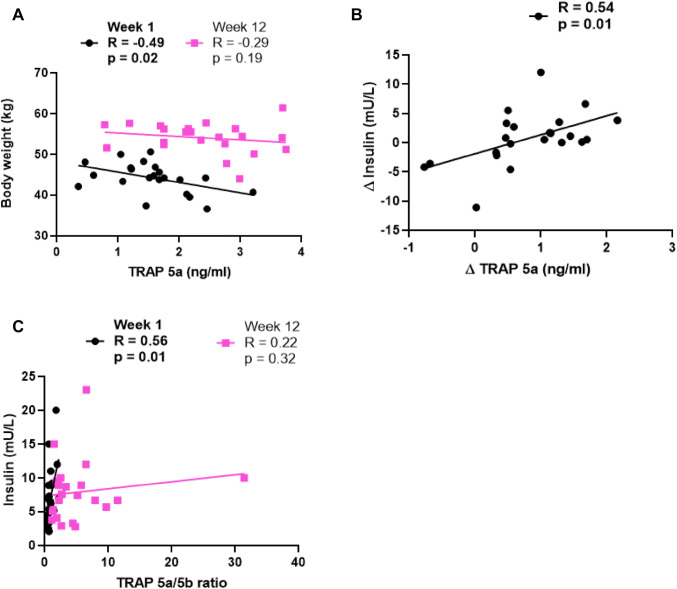
Correlations of TRAP isoforms to weight and insulin. **a** Serum TRAP 5a protein vs body weight. **b** Serum ΔTRAP 5a vs Δinsulin. **c** Serum TRAP 5a/5b ratio vs insulin. *N* = 22 in all analysis

At start, serum TRAP 5a correlated negatively to weight (*R* = − 0.49, *p* = 0.02 (Table 1; Fig. 2a). However, serum TRAP 5a and TRAP 5b did not correlate with most of the investigated parameters (BMI, total fat mass, and total lean mass; Table 1). Additionally, changes in biochemical parameters related to metabolism, i.e., insulin, correlated to the observed changes in TRAP 5a and TRAP 5a/5b, respectively, i.e., Δinsulin to ΔTRAP 5a; *R* = 0.54, *p* = 0.01; TRAP 5a/5b to insulin at week 1; *R* = 0.56, *p* = 0.01, respectively (Table 1, Fig. 2b, c).

**Table 1:** Correlations of TRAP isoforms to weight and adipose tissue metabolism parameters

	TRAP isoform	Week 1		Week 12		Delta	
		*p* value	*R* value	*p* value	*R* value	*p* value	*R* value
Weight (kg)	5a	0.02*	− **0.49**	0.19	− 0.29	0.64	0.11
BMI (kg/m^2^)	5a	0.21	− 0.28	0.95	− 0.01	0.77	0.07
Total fat mass (%)	5a	0.53	− 0.14	0.12	− 0.34	0.76	0.07
Fat mass (g)	5a	0.43	− 0.18	0.31	− 0.23	0.95	0.02
Total lean mass (g)	5a	0.09	− 0.37	0.89	0.03	0.54	− 0.11
Total adiponectin (mg/L)	5a	0.97	− 0.01	0.46	0.17	0.37	0.20
Insulin mU/L)	5a	0.53	0.14	0.93	0.02	0.01**	**0.54**
Weight (kg)	5b	0.58	− 0.13	0.25	− 0.26	0.97	0.01
BMI (kg/m^2^)	5b	0.51	0.15	0.93	0.02	0.85	− 0.04
Total fat mass (%)	5b	0.50	− 0.15	0.97	0.01	0.92	− 0.02
Fat mass (g)	5b	0.47	− 0.16	0.92	− 0.02	0.82	0.05
Total lean mass (g)	5b	0.74	0.08	0.77	− 0.07	0.97	0.01
Total adiponectin (mg/L)	5b	0.19	− 0.29	0.85	− 0.04	0.06	− 0.41
Insulin (mU/L)	5b	0.11	− 0.35	0.79	− 0.06	0.21	− 0.28
Weight (kg)	5a/5b	0.25	− 0.26	0.41	0.19	0.53	0.14
BMI (kg/m^2^)	5a/5b	0.54	− 0.14	0.67	0.10	0.61	0.12
Total fat mass (%)	5a/5b	0.41	0.19	0.89	− 0.03	0.15	0.32
Fat mass (g)	5a/5b	0.41	0.19	0.95	0.02	0.14	0.33
Total lean mass (g)	5a/5b	0.08	− 0.38	0.67	0.10	0.51	0.15
Total adiponectin (mg/L)	5a/5b	0.06	0.41	0.66	0.10	0.75	0.07
Insulin mU/L)	5a/5b	0.01**	**0.56**	0.32	0.22	0.30	0.23

Absolute values for adipose tissue metabolism parameters can be found in [28, 29] (previously published parameters) and in Supplementary Table I É(previously unpublished parameters)

Bold values indicate that are statistically significant. All analysis *n* = 22. *p < 0.05, **p < 0.01

### TRAP 5a, TRAP 5b and TRAP 5a/5b and their changes correlate to bone parameters

**Fig. 3:**
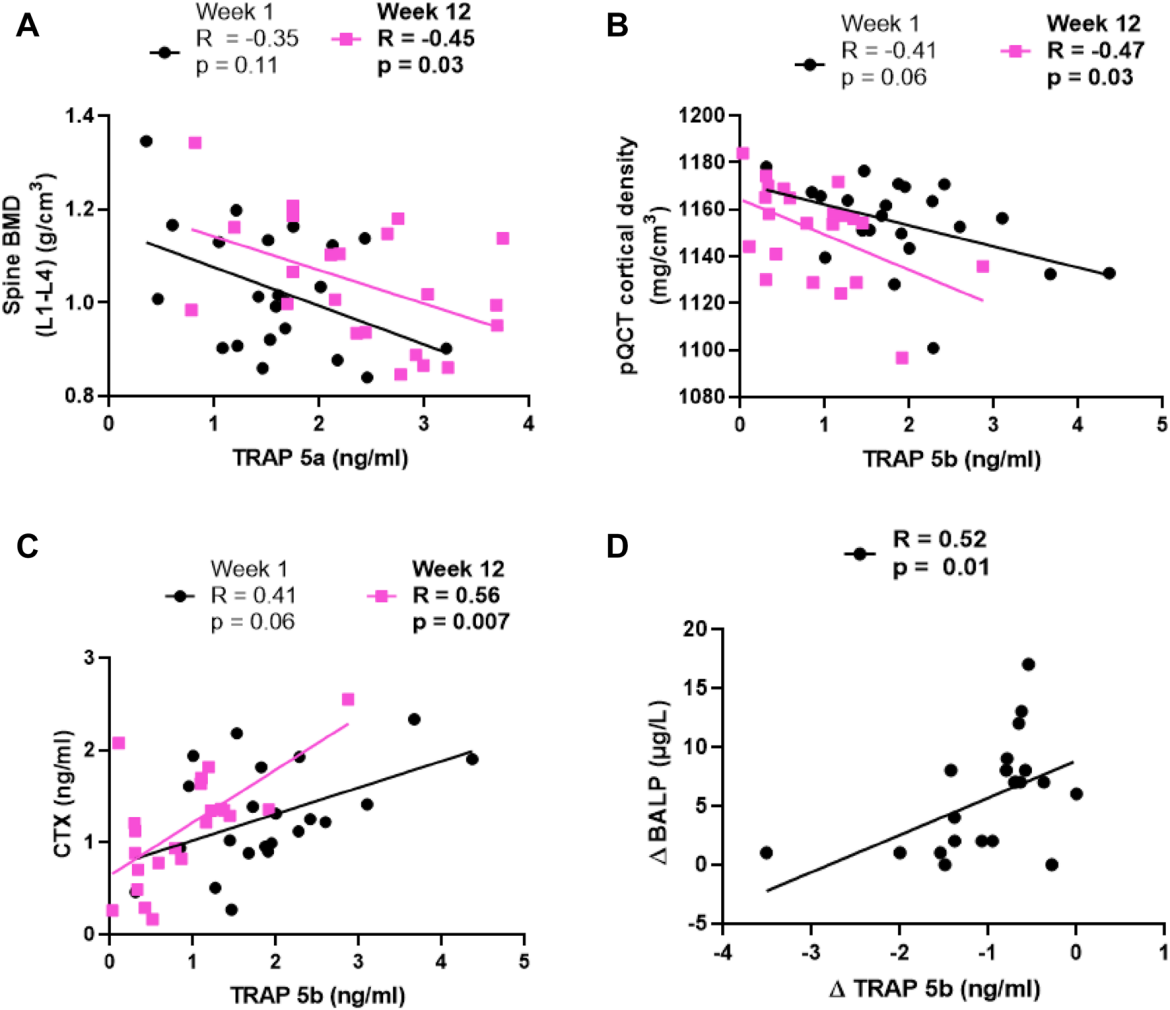
Correlations of TRAP isoforms to bone mass and biochemical parameters. **a** Serum TRAP 5a protein vs spine BMD. **b** Serum TRAP 5b protein vs pQCT cortical bone density. **c** Serum TRAP 5b protein vs CTX. **d** Serum ΔTRAP 5b protein vs ΔBALP. *N* = 22 in all analysis

TRAP 5a correlated inversely to lumbar spine (L1-L4) BMD at week 12, but not at the study start; *R* = − 0.45, *p* = 0.03; *R* = − 0.35, *p* = 0.11, respectively (Table 2, Fig. 3a). Δ lumbar spine (L1-L4) BMC was negatively correlated to ΔTRAP 5a (*R* = − 0.49, *p* = 0.02) and had a tendency for inverse correlation both at week 1 and week 12 (*R* = − 0.41, *p* = 0.06; *R* = − 0.42, *p* = 0.05, respectively). TRAP 5b at week 1 correlated to calcaneal BMD and trabecular density measured by pQCT (*R* = − 0.46, *p* = 0.03; *R* = − 0.54, *p* = 0.01, respectively). At week 12, TRAP 5b correlated positively to cortical density (Fig. 3b, *R* = − 0.47, *p* = 0.03). Changes in trabecular density were positively correlated to changes in TRAP 5b (*R* = 0.50, *p* = 0.02).

**Table 2:** Correlations of TRAP isoforms to bone mass and biochemical parameters

	TRAP isoform	Week 1		Week 12		Delta	
		*p* value	*R* value	*p* value	*R* value	*p* value	*R* value
Total BMD (g/cm^2^)	5a	0.56	− 0.13	0.19	− 0.27	0.92	0.02
Total BMC (g)	5a	0.17	− 0.30	0.40	− 0.19	0.50	− 0.15
Spine BMD (L1–L4) (g/cm^2^)	5a	0.11	− 0.35	0.03*	− **0.45**	0.06	− 0.40
Spine BMC (L1-L4) (g)	5a	0.06	− 0.41	0.05	− 0.42	0.02*	− **0.49**
Calcaneal BMD (g/cm^2^)	5a	0.84	− 0.05	0.93	0.02	0.88	− 0.03
Calcaneal BMC (g)	5a	0.47	− 0.16	0.44	− 0.18	0.47	− 0.16
pQCT cortical density (mg/cm^3^)	5a	0.92	− 0.02	0.35	− 0.21	0.34	0.21
pQCT trabecular density (mg/cm^3^)	5a	0.73	− 0.08	0.31	− 0.23	0.07	− 0.39
BALP (µg/L)	5a	0.35	0.21	0.73	0.08	0.33	0.22
CTX (ng/ml)	5a	0.92	− 0.02	0.57	0.13	0.43	0.18
Total osteocalcin (ng/mL)	5a	0.63	0.11	0.74	0.08	0.12	0.34
Undercarboxylated osteocalcin (ng/mL)	5a	0.39	0.19	0.77	0.07	0.14	0.32
Carboxylated osteocalcin (ng/mL)	5a	0.38	− 0.20	0.91	0.02	0.97	− 0.00
25OH vitamin D (nmol/L)	5a	0.73	0.08	0.81	0.06	0.31	0.23
Total BMD (g/cm^2^)	5b	0.38	0.20	0.45	− 0.17	0.30	0.23
Total BMC (g)	5b	0.34	0.21	0.57	− 0.13	0.46	0.17
Spine BMD (L1–L4) (g/cm^2^)	5b	0.80	0.06	0.13	− 0.33	0.97	0.01
Spine BMC (L1–L4) (g)	5b	0.99	− 0.00	0.05	− 0.42	0.42	0.18
Calcaneal BMD (g/cm^2^)	5b	0.03*	**0.46**	0.14	0.32	0.14	0.32
Calcaneal BMC (g)	5b	0.09	0.37	0.77	0.07	0.04*	**0.44**
pQCT cortical density (mg/cm^3^)	5b	0.06	− 0.41	0.03*	− **0.47**	0.19	0.29
pQCT trabecular density (mg/cm^3^)	5b	0.009**	**0.54**	0.54	0.14	0.02*	**0.50**
BALP (µg/L)	5b	0.93	− 0.02	0.19	0.29	0.01*	**0.52**
CTX (ng/mL)	5b	0.06	0.41	0.007**	**0.56**	0.18	0.30
Total osteocalcin (ng/mL)	5b	0.58	0.13	0.06	0.41	0.19	0.29
Undercarboxylated osteocalcin (ng/mL)	5b	0.94	0.02	0.16	0.31	0.53	− 0.14
Carboxylated osteocalcin (ng/mL)	5b	0.71	0.08	0.60	0.12	0.28	0.24
25OH vitamin D (nmol/L)	5b	0.73	− 0.08	0.76	− 0.07	0.47	− 0.16
Total BMD (g/cm^2^)	5a/5b	0.32	− 0.22	0.50	0.15	0.29	0.24
Total BMC (g)	5a/5b	0.05*	− **0.43**	0.64	0.11	0.51	0.15
Spine BMD (L1–L4) (g/cm^2^)	5a/5b	0.28	− 0.24	0.21	0.28	0.87	− 0.04
Spine BMC (L1–L4) (g)	5a/5b	0.20	− 0.28	0.13	0.34	0.70	− 0.09
Calcaneal BMD (g/cm^2^)	5a/5b	0.02*	− **0.50**	0.06	− 0.41	0.26	0.25
Calcaneal BMC (g)	5a/5b	0.004**	− **0.58**	0.30	− 0.23	0.36	0.20
pQCT cortical density (mg/cm^3^)	5a/5b	0.01**	**0.53**	0.03*	**0.47**	0.45	0.17
pQCT trabecular density (mg/cm^3^)	5a/5b	0.04*	− **0.45**	0.37	− 0.20	1.00	− 0.00
BALP (µg/L)	5a/5b	0.20	0.28	0.30	− 0.23	0.50	− 0.15
CTX (ng/mL)	5a/5b	0.003**	− **0.60**	0.01**	− **0.57**	0.06	− 0.41
Total osteocalcin (ng/mL)	5a/5b	0.71	0.09	0.10	− 0.36	0.14	− 0.33
Undercarboxylated osteocalcin (ng/mL)	5a/5b	0.10	0.36	0.46	− 0.17	0.53	− 0.14
Carboxylated osteocalcin (ng/mL)	5a/5b	0.58	− 0.12	0.95	− 0.13	0.92	0.02
25OH vitamin D (nmol/L)	5a/5b	0.76	0.07	0.79	− 0.06	0.40	0.19

Absolute values for bone mass and biochemical parameters can be found in [28, 29] (previously published parameters) and in Supplementary Table I (previously unpublished parameters)

Bold values indicate that are statistically significant. All analysis *n* = 22. *p < 0.05, **p < 0.01

TRAP 5a/5b correlated to total BMC (*R* = − 0.43, *p* = 0.05), calcaneal BMD (*R* = − 0.50, *p* = 0.02), calcaneal BMC (*R* = − 0.58, *p* = 0.004), cortical density (R = 0.53, p = 0.01) and trabecular density (*R* = − 0.45, *p* = 0.04) (Table 2).

TRAP 5a and TRAP 5b were analyzed for correlation to the bone formation marker BALP and the bone resorption marker CTX. Serum TRAP 5a levels were not correlated to the above-mentioned parameters (Table 2). TRAP 5b showed a tendency for correlation with carboxy-terminal cross-linking telopeptide of type I collagen (CTX) at the study start and correlated positively at week 12 (*R* = 0.41, *p* = 0.06; (Fig. 3c) *R* = 0.56, *p* = 0.01, respectively). TRAP 5a/5b correlated negatively to CTX at both week 1 (*R* = − 0.60, *p* = 0.003) and week 12 (*R* = − 0.57, *p* = 0.02). Moreover, TRAP 5b changes correlated positively to changes in BALP (ΔTRAP 5b versus ΔBALP, (Fig. 3d) *R* = 0.52, *p* = 0.01).

## Discussion

In this prospective interventional study, we investigated changes in the circulating levels of TRAP 5a and TRAP 5b derived from patients with severe AN during rapid weight gain therapy for 12 weeks. Markers of bone turnover, including TRAP 5b, have previously been studied and assessed for their potential to predict the uncoupling of bone formation and resorption in patients with AN [23, 24]. The present study constitutes, to our knowledge, the first effort to address changes in both serum TRAP 5a and TRAP 5b during large metabolic changes over short period of time in patients that initially are in a late stage of severe AN and return to a more normalized BMI. Moreover, this is also the first study to investigate the potential use of TRAP 5a and TRAP 5b, and the 5a/5b ratio, as markers to reflect restoration of bone remodeling in patients with AN during nutritional therapy resulting in a weight increase mainly due to substantial net gain in fat mass. Patients were served six meals daily according to the described nutritional plan, which resulted in a large change in body composition. Fat mass percentage was highly increased from 11.4 to 26.7%, demonstrating that the BMI increase was mainly due to increased fat mass and not lean mass. Hence, this therapy provides a possible clinical model to study changes of the different forms of TRAP within a rather short period of time during large changes in energy metabolism.

Serum TRAP 5a was upregulated after 12 weeks and has previously been shown to be associated with adipose parameters [12, 30] and bone tissue [31]. Serum TRAP 5a reached levels that were in the same magnitude that has been reported for healthy individuals [32], which also is in concurrence with bone and weight assessments reaching a near-healthy state [3]. The lack of association between TRAP 5a or TRAP 5a/5b and anthropometric fat parameters, e.g., BMI, fat mass and fat content does, however, not support a role for TRAP 5a in expansion of the adipose tissue in this particular situation, in contrast to the development of obesity [12]. A major contributor may be related to whether the accumulation of adipose tissue occurs as a result of going from underweight to normal as in this cohort or pathological, i.e., from normal to obese weight gain [12]. Weight gain in AN versus obesity reflects different states with different components such as adipose inflammation and metabolic aberrations that could affect the involvement of TRAP 5a. On the other hand, the observed downregulation of TRAP 5b is in accordance with weight restoration and increased bone mass after weight gain therapy in the AN group [6]. Thus, the increased bone mass is most likely due to a combination of increased bone formation, as reflected by the increase in bone anabolic parameters, i.e., levels of BALP induced by the 12-week therapy [6], as well as decreased bone resorption and lower numbers of osteoclasts, as reflected by decreased serum levels of TRAP 5b.

The observed changes of TRAP 5a and TRAP 5b (in opposite directions) agree with that of total TRAP (i.e., the sum of TRAP 5a and 5b was not significantly changed). The TRAP 5a/TRAP 5b ratio was also significantly altered with approximately a 4-fold increase). The abundance of TRAP 5a, that is, 4-fold more than TRAP 5b, has previously been reported in healthy individuals where TRAP 5a has been in the range of 4-8 ng/ml [33, 34], while other studies have reported TRAP 5b levels in the range of 1–3 ng/ml [23, 24]. Thus far, there are no studies that have investigated the ratio between TRAP 5a and 5b directly in the same serum sample from humans. Previously, it has been reported that TRAP 5a is secreted by macrophages from adipose tissue [30]; thus, the observed inverse correlation between TRAP 5a and weight at baseline, but not at week 12, could be due to exceptionally low amounts of adipose tissue and increased inflammation at the study start.

Interestingly, TRAP 5a was negatively associated with bone parameters such as lumbar spine BMD, which further implicate the involvement of TRAP 5a in bone regulation since it has been proposed to function as both positive and negative regulators of, e.g., osteoblasts [35]. However, TRAP 5a did not correlate to most of the bone parameters measured in this study. The role of TRAP 5a as a bone marker is unknown and this study is limited by a rather small number of participants; therefore, it is difficult to conclude the reason and implication for this. Future studies including larger cohorts are necessary to elucidate the possible role of TRAP 5a as a bone marker in patients with AN or other pathologies affecting bone.

TRAP 5b was associated with bone parameters such as CTX, calcaneal BMD and BMC, as well as cortical and trabecular density, in accordance with previous studies [19], thus further demonstrating that serum TRAP 5b is predominantly derived from bone tissue and reflects the number and activity of osteoclasts. The correlation of ΔTRAP 5b to ΔpQCT trabecular density is additionally consistent with more rapid turnover in trabecular compared to cortical bone. It is difficult to reason why TRAP 5b is correlated to, e.g., calcaneal BMD but not spinal or total BMD. One reason might be the small number of participants and other that there are site-specific differences in osteoclast metabolism. As for TRAP 5a, future studies including larger cohorts are necessary to elucidate the precise role of TRAP 5b as a bone marker in AN or other bone pathologies.

Additionally, this study further suggests that it is important to calculate the TRAP 5a/5b ratio. TRAP 5a/5b ratio correlated negatively to total BMC and calcaneal BMD/BMC. This strengthens the idea that TRAP correlation to BMD and BMC in spine vs calcaneal bone might be a consequence of site-specific differences between spine and calcaneal bone since TRAP 5a/5b ratio correlates with calcaneal but not spine BMC and BMD at week 1. TRAP 5a/5b ratio also correlates to both cortical (positive) and trabecular (negative) density at week 1 implying that TRAP as a bone marker might not only be site specific between different bones entities but also site specific within a bone. Together, the increased number of bone parameters correlating to TRAP 5a/5b ratio compared to TRAP 5b alone suggests that the TRAP 5a/5b ratio could be a more sensitive parameter for bone resorption than TRAP 5b alone indicating that a low TRAP 5a/5b ratio could predict a pathological bone phenotype.

The observed delta values in serum TRAP 5a, between baseline and 12 weeks and TRAP 5a/5b ratio at week 1, were correlated to changes in serum insulin indicating that TRAP 5a is involved in metabolic changes, which is in conjunction with previous reports [33, 36]. However, the exact mechanism by which TRAP 5a responds to metabolic changes and insulin sensitivity/resistance remains to be elucidated. Finally, Δ decrease in TRAP 5b was shown to be correlated to Δ increase in BALP, further suggesting that coupling between bone formation and bone resorption is restored during weight gain treatment of the AN cohort.

The rather small number of participants is a limitation, which makes our results difficult to generalize. However, this 12-week nutrition therapy for AN is highly staff-intensive (with 24-h surveillance), which made it difficult to include further AN patients. The lack of a control group is also a limitation; however, controls were not included because we found it unethical to recruit normal-weight young women that would agree to a diet with the objective of gaining weight in a hospitalized environment for 12 weeks.

In conclusion, patients with AN, who received the described intensive weight gain therapy and increased their BMI and body weight, demonstrated also changes, in opposite directions, for serum TRAP 5a and TRAP 5b toward normal levels. The observed decrease for TRAP 5b was linked to increased bone formation and decreased bone resorption, leading to improved bone mass. The increase for TRAP 5a seems to derive from overall systemic changes in bone as well as metabolic changes but is not directly correlated to expansion of adipose tissue. The combination of serum TRAP 5a and TRAP 5b, as well as the restoration of TRAP 5a/TRAP 5b ratio, could be further indicative of reduced bone resorption and overall improved bone homeostasis after 12-week nutrition therapy. Future studies should aim to verify these results in larger cohorts of AN patients as well as other adipose tissue and bone pathologies to fully understand the role of TRAP isoforms as biomarkers.

## Electronic supplementary material

Below is the link to the electronic supplementary material.

Supplementary material 1 (DOCX 21 kb)

Supplementary material 2 (DOCX 56 kb)

## Data Availability

The datasets generated during the current study are available from Dr. Diana Swolin-Eide or Dr. Bojan Tubic on reasonable request.
