# Supplementary material for: Intensive weight gain therapy in patients with anorexia nervosa results in improved serum tartrate-resistant acid phosphatase (TRAP) 5a and 5b isoform protein levels
Source: Eat Weight Disord. 2019 Sep 17;25(5):1387–97. doi: 10.1007/s40519-019-00776-8 (PMC7508747; doi:10.1007/s40519-019-00776-8)
Supplement: Supplementary file 1 — Supplementary material 1 (DOCX 21 kb) [file 40519_2019_776_MOESM1_ESM.docx]

**Supplementary table I. Absolute values for previously unpublished parameters.**

Absolute values for previously published parameters are found in [28].

|  | **Week 1** | **Week 12** | **P-value** |
| --- | --- | --- | --- |
| **Weight (kg)** | 44.25 (36.60 - 61.40) | 54.30 (44.00 - 61.40) | **** |
| **BMI (kg/m^2^)** | 15.04 (13.40 – 13.30) | 19.05 (16.20 – 20.60) | **** |

Data is shown as median (minimum – maximum). All analysis n = 22. * = 0.05, **** = <0.0001.
