# Supplementary material for: Intensive weight gain therapy in patients with anorexia nervosa results in improved serum tartrate-resistant acid phosphatase (TRAP) 5a and 5b isoform protein levels
Source: Eat Weight Disord. 2019 Sep 17;25(5):1387–97. doi: 10.1007/s40519-019-00776-8 (PMC7508747; doi:10.1007/s40519-019-00776-8)

Supplementary figure 1.

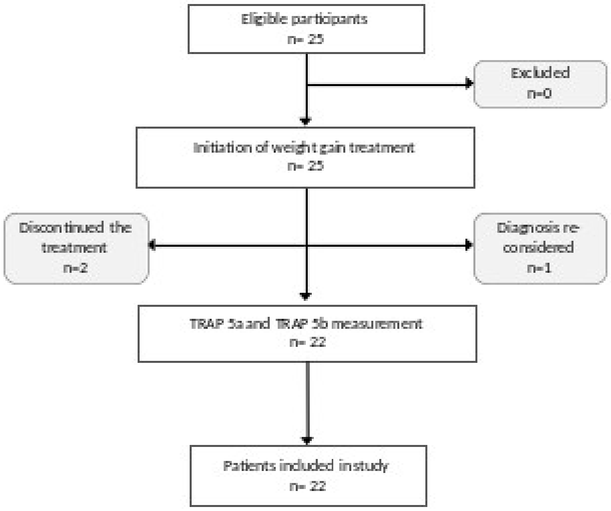

Supplement: Supplementary file 2 [file 40519_2019_776_MOESM2_ESM.docx]
